# Multifunctional Magneto-Plasmonic Fe_3_O_4_/Au Nanocomposites: Approaching Magnetophoretically-Enhanced Photothermal Therapy

**DOI:** 10.3390/nano11051113

**Published:** 2021-04-25

**Authors:** Iuliia Mukha, Oksana Chepurna, Nadiia Vityuk, Alina Khodko, Liudmyla Storozhuk, Volodymyr Dzhagan, Dietrich R.T. Zahn, Vasilis Ntziachristos, Andriy Chmyrov, Tymish Y. Ohulchanskyy

**Affiliations:** 1 Chuiko Institute of Surface Chemistry, National Academy of Sciences of Ukraine, 03164 Kyiv, Ukraine; nvityuk@gmail.com (N.V.); l.storozhuk@ucl.ac.uk (L.S.); 2 College of Physics and Optoelectronic Engineering, Shenzhen University, Shenzhen 518060, China; ok.chepurna@gmail.com; 3 Institute of Physics, National Academy of Sciences of Ukraine, 03028 Kyiv, Ukraine; khodkoalina@gmail.com; 4 Department of Physics & Astronomy, University College London Healthcare Biomagnetic and Nanomaterials Laboratory, London W1S 4BS, UK; 5 V. Lashkaryov Institute of Semiconductors Physics, National Academy of Sciences of Ukraine, 03028 Kyiv, Ukraine; dzhagan@isp.kiev.ua; 6 Semiconductor Physics, Chemnitz University of Technology, 09107 Chemnitz, Germany; zahn@physik.tu-chemnitz.de; 7 Helmholtz Zentrum München, Institute of Biological and Medical Imaging, 85764 Neuherberg, Germany; v.ntziachristos@tum.de (V.N.); andriy.chmyrov@helmholtz-muenchen.de (A.C.); 8 School of Medicine, Technical University of Munich, 80333 Munich, Germany

**Keywords:** magneto-plasmonic, Fe_3_O_4_ and Au nanocomposites, magnetophoretic control, photothermal therapy, near infrared, microbubbles, HeLa cells

## Abstract

Magneto-plasmonic nanocomposites can possess properties inherent to both individual components (iron oxide and gold nanoparticles) and are reported to demonstrate high potential in targeted drug delivery and therapy. Herein, we report on Fe_3_O_4_/Au magneto-plasmonic nanocomposites (MPNC) synthesized with the use of amino acid tryptophan via chemical and photochemical reduction of Au ions in the presence of nanosized magnetite. The magnetic field (MF) induced aggregation was accompanied by an increase in the absorption in the near-infrared (NIR) spectral region, which was demonstrated to provide an enhanced photothermal (PT) effect under NIR laser irradiation (at 808 nm). A possibility for therapeutic application of the MPNC was illustrated using cancer cells in vitro. Cultured HeLa cells were treated by MPNC in the presence of MF and without it, following laser irradiation and imaging using confocal laser scanning microscopy. After scanning laser irradiation of the MPNC/MF treated cells, a formation and rise of photothermally-induced microbubbles on the cell surfaces was observed, leading to a damage of the cell membrane and cell destruction. We conclude that the synthesized magneto-plasmonic Fe_3_O_4_/Au nanosystems exhibit magnetic field-induced reversible aggregation accompanied by an increase in NIR absorption, allowing for an opportunity to magnetophoretically control and locally enhance a NIR light-induced thermal effect, which holds high promise for the application in photothermal therapy.

## 1. Introduction

Gold nanoparticles (including nanoshells, nanorods, nanostars, etc.) represent a well-known class of photoactive nanoagents that can be applied in photothermal therapy (PTT) of cancer and other diseases [1,2,3,4]. Due to the phenomenon of localized surface plasmon resonance (LSPR) [5], spherical gold nanoparticles (NPs) in colloidal suspensions display a specific red color that corresponds to a characteristic absorption band peaked at ~520–530 nm. The advantages of plasmonic gold nanospheres over anisotropic nanostructures comprise generally simple synthesis, narrow size distribution and, especially, higher thermodynamic stability [6]. Gold NPs-based phototherapeutic agents were demonstrated to produce efficient PTT effects both in vitro and in vivo [1,2,3,4]. The LSPR band position can be shifted upon association (aggregation) of gold NPs: It leads to the formation of a new absorption band at longer wavelengths as a result of electric dipole-dipole interaction and coupling between the plasmons of neighboring particles in the formed aggregates [7,8,9,10]. In turn, a shift of the absorption of the aggregated gold nanoparticles towards the near-infrared (NIR) region allows for utilization of NIR light (which is known to deeper penetrate biological tissues due to lesser absorption and scattering) for induction or enhancement of photothermal effects [11]. On the other hand, NIR plasmonic absorbance for photothermal therapy can be achieved by using anisotropic gold nanoparticles (e.g., nanorods, nanocages, nanoshells, nanostars, etc. [4,12,13], or hybrid (composite) nanostructures that comprise gold with other materials [14,15,16]. Due to higher intrinsic NIR absorbance and photothermal transfer ability of gold-based anisotropic nanoparticles, they showed promising preliminary results in PTT applications. However, they are relatively difficult to synthesize, can be contaminated with toxic surfactants used in their preparation (e.g., cetyltrimethylammonium bromide, CTAB) and are also relatively large in size (~over 50 nm), which can promote an efficient capture by the reticuloendothelial system (RES), limiting systemic circulation time, tumor targeting, efficient endocytosis into cancerous cells and posing potential clearance problems when clinically tried [11,17,18]. Recent reports suggest that these disadvantages of PTT with complex anisotropic gold nanostructures can be overcome through an employment of the gold NPs with aggregation triggered by tumor-specific stimuli (e.g., pH), thus inducing NIR absorbance in situ in the targeted area (i.e., malignant tissue) and providing significant improvement of PTT of cancer [19,20]. 

Currently, there is a growing interest towards nanocomposites of iron oxide (Fe_3_O_4_) and gold (Au), which combine magnetic and optical (plasmonic) properties inherent to both individual components; such magneto-plasmonic nanocomposites manifest high potential for targeted drug delivery and therapeutic applications [21,22]. In the case of complex magneto-plasmonic nanosystems, a magnetic field can be applied for their targeted delivery to cancer cells and increased uptake, allowing for enhanced photothermal effect through irradiation in the plasmonic absorption band, followed by efficient PTT demonstrated in vitro and in vivo [23,24,25]. On the other hand, the application of a magnetic field can induce interaction or aggregation of magneto-plasmonic nanoparticles that may cause changes in their optical properties [26,27]. A number of studies reported magneto-plasmonic nanosystems with magnetically induced aggregation producing change of their optical properties (e.g., enhancing the surface-enhanced Raman spectroscopy (SERS) signal [28,29,30,31]. However, no study devoted to a magnetic field induced shift of the plasmonic absorbance of magneto-plasmonic nanostructures into NIR region could be found. Moreover, to the best of our knowledge, the use of a magnetic field to generate NIR absorption of magneto-plasmonic nanostructures, resulting in a boost in efficiency of NIR induced photothermal effect/PTT, have not been reported before. 

In this work, we report the preparation of Fe_3_O_4_/Au magneto-plasmonic nanocomposites (MPNC) containing nanosized magnetite and gold constituents. The size and morphology of Fe_3_O_4_/Au in colloids with various gold content were characterized by transmission electron microscopy and dynamic light scattering methods, the optical properties were studied by UV–VIS spectroscopy; X-ray photoelectron spectroscopy was applied to assess the elemental composition. Aqueous suspensions of MPNC manifested LSPR absorption band with peak position tunable by the composition of the nanocomposites (i.e., Fe_3_O_4_/Au ratio). Moreover, the absorption of MPNC demonstrated a shift in NIR spectral region and was found to rise due to the reversible aggregation of nanocomposites under an external magnetic field. Even more, a magnetic field induced increase in NIR absorption was shown to correlate with an increase in photothermal effect induced in magnetic field treated MPNC colloids by irradiation with a NIR (808 nm) laser diode. The possibility for therapeutic application of the magnetic field-induced increase in the NIR absorption, which resulted in an enhanced photothermal effect under laser irradiation, was also demonstrated using cancer cells in vitro. Cell membrane damage and cell destruction, which were revealed using confocal laser scanning microscopy, evidently originated from the formation of microbubbles on the cell surface due to the photothermal effect caused by the laser scanning irradiation of MPNC. It should also be noted that our MPNC were found to be core-satellite structures, which may have an advantage of higher light-to-heat conversion efficiency, as it was recently shown [32]. Thus, the synthesized magneto-plasmonic Fe_3_O_4_/Au nanosystems are highly promising for the magnetophoretically-controlled photothermal therapy. The possibility to enhance NIR absorption on demand in situ, in a targeted diseased tissue, to boost the NIR induced photothermal effect can be significant for the field of phototherapy of cancer and other diseases. 

## 2. Materials and Methods

*Chemicals*. Iron(II) chloride tetrahydrate (FeCl_2_∙4H_2_O, Sigma-Aldrich, Gillingham, UK), iron(III) chloride hexahydrate (FeCl_3_∙6H_2_O, Sigma-Aldrich, Gillingham, UK), ammonium hydroxide solution (NH_4_OH, Sigma-Aldrich, Gillingham, UK), tryptophan (C_11_H_12_N_2_O_2_, SC12-20120713, Xintai Jiahe International Co., Ltd., Shandong Province, China), sodium hydroxide (NaOH, Merck, Darmstadt, Germany), tetrachloroauric acid (HAuCl_4_, Merck, Darmstadt, Germany), pluronic F68 ((C_3_H_6_O·C_2_H_4_O)x, Aldrich, Milwaukee, W, USA), sodium citrate (Na_3_C_6_H_5_O_7_, Acros, Renningen, Germany).

### 2.1. Synthesis of Fe_3_O_4_/Au Nanocomposites

Two methods, chemical and photochemical, were used to obtain plasmonic nanosized gold in the presence of nanosized magnetite, pre-prepared according to [33]. In particular, Fe_3_O_4_ nanoparticles were synthesized by coprecipitation of ferric and ferrous chloride salts in a 1:2 molar ratio with ammonium hydroxide under an inert atmosphere at 70 °C. In a simplified manner, the reaction mechanism can be presented as: Fe^2+^ + 2Fe^3+^ + 8OH^−^ ⇆ Fe(OH)_2_ + 2Fe(OH)_3_→Fe_3_O_4_↓ + 4H_2_O. Briefly, 300 mL of 0.38 M ammonium hydroxide solution used as a precipitation agent was mixed with 30 mL of FeCl_2_·4H_2_O (0.02 M) and FeCl_3_·6H_2_O (0.04 M) at a continuous stirring speed of 700 rpm. The obtained nanoparticles were washed by centrifugation (7500 rpm, 10 min, 3 times, 20 mL DIH_2_O) and redispersed in DIH_2_O leading to neutral pH. The obtained powder was stabilized by sodium oleate. 

Colloidal Fe_3_O_4_/Au nanocomposites were prepared in an aqueous solution of tryptophan (Trp) with initial alkaline medium (pH = 10) reached by a 1 N solution of sodium hydroxide using the following concentrations of reagents: C_Trp_ = 2 × 10^−4^ M, C Fe_3_O_4_ = 5 × 10^−5^ M; the synthesis was performed at different Au concentration, C_Au_ varied from 1 × 10^−4^ to 3 × 10^−4^ M. Fe_3_O_4_ was injected in the solution before tetrachloroauric acid.

While the chemical reduction of Au ions by tryptophan proceeded at boiling temperature, the photochemical process occurred at room temperature under UV light irradiation during 5 h using a light emission diode UV-C LED (LGInnotek) with a wavelength of λ = 278 nm and an output optical power density of P = 3.0 ± 0.1 mW/cm^2^ as a light source in the 40 mL quartz glass.

The Fe_3_O_4_/Au nanocomposites formed in the colloid were aggregated in a certain way with nitric acid, accumulated by a commercial NdFeB magnet and washed with distilled water twice. Final Fe_3_O_4_/Au suspensions were dissolved at C_Au_ = 3 × 10^−3^ M in polymer pluronic F68 and sodium citrate for further application. 

### 2.2. Characterization of Fe_3_O_4_/Au Nanocomposites

*Optical properties*. The absorption spectra of the MPNC colloids were recorded in a 1 cm quartz cell using a spectrophotometer Lambda 35 (Perkin-Elmer, Waltham, MA, USA).

*Size and morphology.* Gold and Fe_3_O_4_/Au NPs were characterized by transmission electron microscopy (TEM) using a JEOL-1200 EX (JEOL, Tokyo, Japan) at an accelerating voltage of 120 kV. The NP dispersions were diluted in DI water, dropped onto a carbon-coated copper grid, and dried at room temperature. Particle size analysis was performed from TEM images manually via the image analysis software ImageJ. Particle size distributions (presented in insets for corresponding TEM image) have the following notations: dTEM is the average diameter, δd is the standard deviation, and n is the number of particles counted to obtain the particle size distribution.

*Size and zeta potential distribution*. Distribution of MPNC size and zeta potential was assessed by a dynamic light scattering (DLS) method using laser correlation spectrometer Zeta Sizer Nano S (Malvern Panalytical Ltd, Malvern, UK) equipped with a correlator (multi-computing correlator type 7032 CE). The helium-neon laser LGN–111 was used with the output power of 25 mW and wavelength of 633 nm to irradiate the suspension. The registration and statistical processing of the scattered laser light at 173° from the suspension were performed three times during 120 s at 25 °C. The resulting autocorrelation function was treated with standard computer programs PCS–Size mode v.1.61.

*X-ray photoelectron spectroscopy*. X-ray photoelectron spectroscopy (XPS) measurements were performed with an ESCALAB 250Xi X-ray photoelectron spectrometer microprobe (Thermo Scientific, Waltham, MA, USA) equipped with a monochromatic Al Kα (hν = 1486.68 eV) X-ray source. A pass energy of 200 eV was used for survey spectra and 20 eV for high-resolution core-level spectra (providing a spectral resolution of 0.5 eV). Spectra deconvolution and quantification were performed using the Avantage Data System (Thermo Scientific). The linearity of the energy scale was calibrated by the positions of the Fermi edge at 0.00 ± 0.05 eV, Au4f_7/2_ at 83.95 eV, Ag3d_5/2_ at 368.20 eV, and Cu2p_3/2_ at 932.60 eV measured on in situ cleaned metal surfaces. To prevent charging, the NCs samples were measured employing the built-in charge compensation system. Finally, the spectra were corrected to C 1s sp3 peak at 284.8 eV as the common internal standard for BE calibration [34].

*Photothermal effect*. The PT effect produced by MPNC was assessed using a laser diode emitting at 808 nm with an irradiation power density of 70 mW/cm^2^. During the experiment, a thermocouple connected to a multimeter for temperature measurement was dipped into the cuvette with the irradiated MPNC suspension.

*Optical imaging*. Confocal laser scanning microscopy imaging in fluorescence, reflection and transmission channels was performed using a home-built confocal microscope based on a commercial Olympus IX83 microscope. Fluorescence and reflection imaging were performed with 485 nm laser (LDH-D-C-485, PicoQuant, Berlin, Germany) driven by a corresponding driver (PDL 800-D dual mode, PicoQuant, Berlin, Germany). For heating, a pulsed laser emitting at 593 nm emission wavelength (Katana 06-HP, OneFive, Zürich, Switzerland) was utilized. Both laser beams were combined using a dichoric beam splitter (ZT594DCRB, Chroma Technology Corp, Bellows Falls, VT, USA), collimated and expanded using telescopes in order to fill the back aperture of the UPLSAPO60XW 1.2 NA water immersion objective (Olympus, Tokyo, Japan). The fluorescence from the sample was separated from the excitation beams using a combination of a dichroic mirror (ZT488DCRB) and a bandpass emission filter (ET535/70 m), both from Chroma Technology Corp, USA. After spatial filtering with a 50 µm pinhole (MPH16, Thorlabs, Dachau, Germany) the fluorescence was detected using an avalanche photodiode (SPCM-AQRH-13, Excelitas, Waltham, MA, USA). The reflected light was separated from the illumination light using a 2 µm thin pellicle beamsplitter (BP108, Thorlabs, Dachau, Germany) and redirected towards a PMT module (H10723-01, Hamamatsu Photonics, Hamamatsu City, Japan). The voltage signal from the PMT was digitalized by a DAQ card (PCIe-6353, National Instruments, Austin, TX, USA) and plotted using home-written scripts in MATLAB (Mathworks, Natick, MA, USA), which also controlled the scanning mirrors (dynAXIS 421, ScanLab GmbH, Munich, Germany) scanning the focal spot to image the sample area.

Optical transmission images were acquired using a white light LED lamp and a CCD camera (DMK 23U274, The Imaging Source, Bremen, Germany) coupled to a side port of the microscope.

*Cell culture.* To study the photothermal effect of the prepared Fe_3_O_4_/Au nanocomposites on cancer cells in vitro, the ovarian cancer cell line (HeLa) was used. For better visualization of the cell cytoskeleton in fluorescence imaging, the keratin protein of HeLa cells, which is an intermediate filament protein that forms cytoskeletal filament networks in epithelial cells, was fused with a GFP-like fluorescent protein rsEGFP2 [35]. HeLa cells seeded in 35 mm cell dishes (P35G-0.170-14-C, Mattek, Ashland, MA, USA) were maintained in a DMEM medium with 10% fetal bovine serum (FBS), 100 U/mL penicillin/streptomycin solution and 1 mM sodium pyruvate. Cells were gently rinsed with the pre-warmed to 37 °C buffer (Gibco™ PBS pH 7.4, ThermoFisher, Schwerte, Germany) prior treatment with the MPNC. 

*Demonstration of photothermal effect in vitro.* HeLa cells in a dish, that was placed in a sample holder of the microscope, were incubated with MPNC at C_Au_ = 1.25 × 10^−4^ M under an applied magnetic field for 100 min. Next, HeLa cells treated with Fe_3_O_4_/Au nanocomposites were imaged, with an imaging area of 80 μm × 120 μm. MPNC localization and with cell morphology were monitored in time using transmission, reflection and fluorescence channels of the microscope.

For the induction of bubbles formation, laser irradiation at a wavelength of 593 nm was applied with the following parameters: pulse energy 5 nJ, pulse duration 1 ns, repetition rate 20 MHz, 4 J/cm^2^.

## 3. Results and Discussion

### 3.1. Characterization of Fe_3_O_4_/Au Nanocomposites and Their Optical Properties 

**Figure 1:**
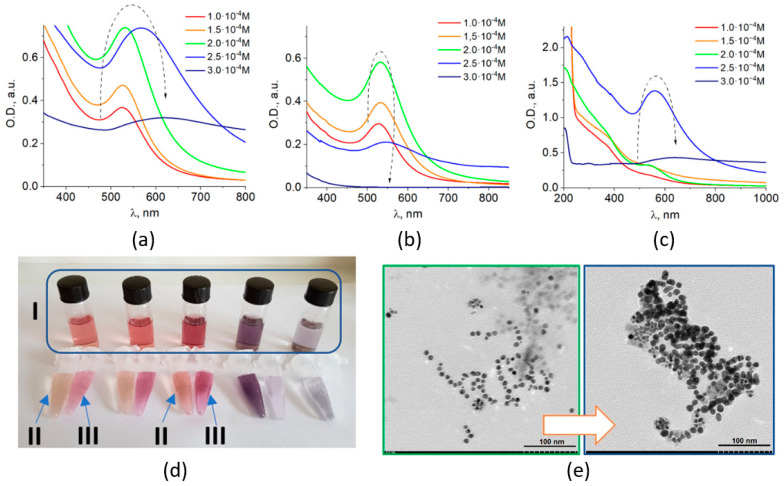
Characterization of Fe_3_O_4_/Au nanocomposites prepared by chemical method. Optical absorption spectra of the initial Fe_3_O_4_/Au colloids with the different gold content (**a**), and their magnetic (**b**) and non-magnetic (**c**) fractions, separated by a magnet. All three samples are shown in (**d**): initial Fe_3_O_4_/Au colloids (I), their magnetic (II) and non-magnetic (III) fractions. TEM images of prepared samples with C_Au_ = 2 × 10^−4^ M (**left**) and C_Au_ = 2.5 × 10^−4^ M (**right**) are shown in (**e**), scale bar 100 nm.

MPNC presented in this work comprised Fe_3_O_4_ and Au constituents. They were prepared via reduction of Au(III) from HAuCl_4_ by amino acid tryptophan (according to a previously developed procedure for the preparation of plasmonic metal colloids with the use of tryptophan [36]) in the presence of pre-formed nanosized magnetite. This approach allowed for obtaining of colloidal Fe_3_O_4_/Au MPNC that exhibit magnetic and optical (plasmonic) properties inherent to both individual components. Optical properties (Figure 1a–c) and stability of nanocomposites (Figure 1d) were found to significantly depend on the metal content.

The synthesized Fe_3_O_4_/Au colloids can be efficiently separated into magnetic and non-magnetic fractions by applying a magnet for each sample (Figure 1d). Hence, the optical absorption spectra of the prepared colloids (Figure 1a) were the sum of those of both magnetic (Figure 1b) and non-magnetic (Figure 1c) components. The LSPR bands of both fractions changed with an increase in gold content in the same manner: increased intensity—shifted maximum—decreased intensity. 

*Initial Fe_3_O_4_/Au colloids.* The intensity of the plasmon absorption band of nanosized gold increased with an increase in metal content from 1 × 10^−4^ to 2 × 10^−4^ M, which was accompanied with a shift of the LSPR peak from 524 to 532 nm. When a higher concentration of 2.5 × 10^−4^ M was used, the LSPR band maximum drastically shifted to ~567 nm. Further increase of the gold content to 3 × 10^−4^ M caused a shift of the LSPR peak to 619 nm, with a concomitant broadening and decrease in plasmon band intensity (Figure 1a).

*Non-magnetic component.* In the separated non-magnetic fraction of MPNC (i.e., gold NPs), the intensity of the LSPR band increased with an increase in C_Au_ from 1 × 10^−4^ to 2 × 10^−4^ M, simultaneously with a shift of the band maximum from 527 to 532 nm (which is typical for the Au/Trp system (data not shown). Further increase in the gold content up to 2.5 × 10^−4^ M caused an aggregation of the particles reflected in the low intense and broad plasmon band with λ_max_ = 546 nm in the absorption spectrum. No formation of the individual gold NPs was observed in the Fe_3_O_4_/Au system with C_Au_ of 3 × 10^−4^ M. 

*Magnetic component.* The plasmon absorption band of Au in the magnetic Fe_3_O_4_/Au component initially appeared as a small shoulder in the absorption spectrum and then rose as a well-shaped band with maximum at 525 nm at C_Au_ = 2 × 10^−4^ M. Similarly, to absorption spectrum of gold NPs at C_Au_ = 2.5 × 10^−4^ M, the plasmon band of the magnetic Fe_3_O_4_/Au component was shifted to 560 nm, but exhibited much higher intensity, with significant absorption in the region of longer wavelengths. Further increase of the gold content caused total aggregation of the particles and the colloid became unstable. Nevertheless, even at C_Au_ = 3 × 10^−4^ M, MPNC still possess a distinct plasmon band with a maximum at 619 nm (Figure 1c).

Thus, the evolution of the optical absorption spectra has the same tendency for both magnetic and non-magnetic fractions. The sharp shift of the plasmon band position and further decreased of its intensity can be attributed to the plasmon coupling [7,8,9,10] caused by interaction (or aggregation) of gold NPs as shown below.

As it follows from the TEM results, nanoparticles in all obtained Fe_3_O_4_/Au systems were mostly spherical with an average size around 10 nm (Figure 1e). For the subsequent samples prepared with C_Au_ = 2 × 10^−4^ M and C_Au_ = 2.5 × 10^−4^ M the nanoparticle diameter was found to be d = 8.9 ± 1.4 nm and d = 10.6 ± 2.7 nm, correspondingly. Thus, the shift of the LSPR band maximum of 35 nm, as expected, is not associated with significant particle growth, but rather with the aggregation of the nanoparticles, as suggested by TEM data (Figure 1e).

**Figure 2:**
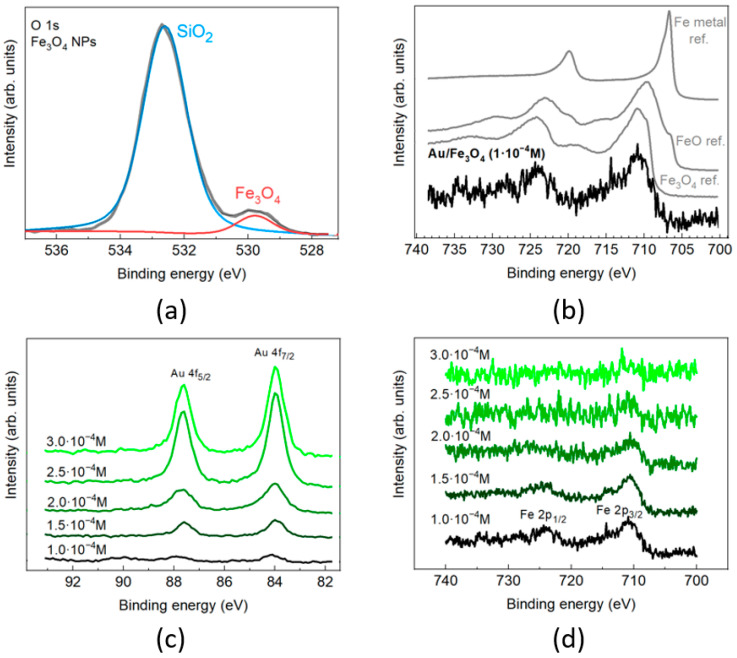
XPS characterization of Fe_3_O_4_/Au nanocomposites. High-resolution O 1s XPS spectra of pure Fe_3_O_4_ NPs sample in the range of Fe 2p orbitals (**a**); high-resolution Fe 2p XPS spectrum of the Fe_3_O_4_/Au (C_Au_ = 1 × 10^−4^ M) in comparison with the spectra of reference samples of Fe_3_O_4_, FeO, and metal Fe from the Avantage database (Thermo Scientific) (**b**); high-resolution XPS spectra of the series of magnetic fraction of Fe_3_O_4_/Au colloids in the range of Au 4f (**c**) and Fe 2p (**d**) peaks.

X-ray photoelectron spectroscopy (XPS) was applied to reveal the composition in of Fe_3_O_4_/Au magnetic components in a series of MPNC colloids. In the spectrum of a control sample of pure magnetite NPs deposited on a Si/SiO_2_ substrate (Figure 2a), the O 1s peak of the Fe_3_O_4_ (BE = 529.8 eV) was well distinguished from the SiO_2_ peak (BE = 532.6 eV) and allowed the Fe:O ratio to be calculated precisely (2%). It amounts to 43:57, i.e., with a good precision coincides with the stoichiometry of Fe_3_O_4_. The spectral line shape of the Fe 2p in a sample prepared with C_Au_ = 1 × 10^−4^ M indicates the initial Fe_3_O_4_ oxide in Fe_3_O_4_/Au (Figure 2b) (direct evaluation of the Fe:O ratio for these samples cannot be performed with sufficient accuracy because of the overlap between oxygen signals from amino acid and SiO_2_ surface of the substrate).

The Au:Fe ratio in the nanocomposites (calculated from the ratio of the corresponding XPS peaks intensities using Avantage software) increased with an increase in Au concentration. The evolution of the absolute and relative intensities of Au 4f and Fe 2p peaks within the series (Figure 2c,d, Table 1) confirms the formation and gradual growth of gold shell on the surface of the magnetite particles. Similarly, to UV–VIS absorption spectra, a drastic difference in XPS spectra between systems with C_Au_ = 2 × 10^−4^ M and C_Au_ = 2.5 × 10^−4^ M was noticed. The Au:Fe ratio in the surface layer of Fe_3_O_4_/Au is the same for the two higher concentrations, thus a saturation of magnetite surface with gold NPs appears to occur.

As Trp is known to absorb light in the UV region, UV light can be used for the activation of the redox reaction between the metal cation (or complex anion [AuCl_4_]^−^) and Trp) It means that not only chemical method, but also photochemical one can be utilized for the Trp-induced preparation of M PNCs. When the chemical method is used, such experimental parameters as initial concentrations, ratio of the reagents and temperature of the reaction can be varied. In the case of the photochemical method, parameters like the irradiation power density and time can be additionally established to control the morphology of plasmonic nanoparticles [37,38]. 

For colloids of Fe_3_O_4_/Au nanocomposites obtained photochemically, we observed similar changes in the optical properties as for the chemically obtained series, but photochemically obtained colloids were overall more stable. The rate of formation of gold NPs under UV irradiation without heating was much slower, as the mechanism of particle growth was apparently affected. This approach contributed to the formation of structures of particles through the amino acid cross-linking.

**Figure 3:**
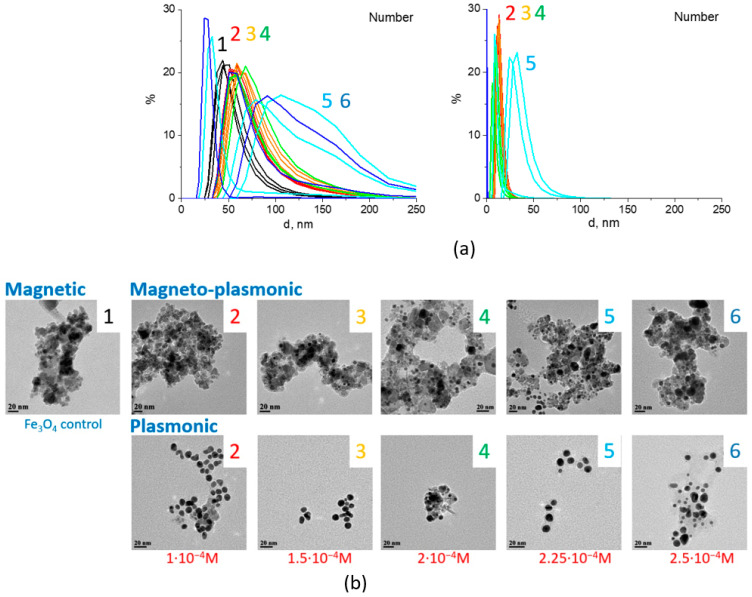
Size assessment for Fe_3_O_4_/Au nanocomposites prepared by photochemical method. DLS size distribution by number basis of magnetic fraction of Fe_3_O_4_/Au colloids, where 1 corresponds to Fe_3_O_4_ control sample, 2 to Fe_3_O_4_/Au prepared using C_Au_ = 1 × 10^−4^ M, 3—1.5 × 10^−4^ M, 4—2 × 10^−4^ M, 5—2.25 × 10^−4^ M, 6—C_Au_ = 2.5 × 10^−4^ M (**a**). TEM images (scale bar 20 nm) of photochemically obtained colloids and their magnetic and non-magnetic fractions are shown in (**b**).

The magnetic component both in the photochemically and chemically prepared samples were separated from colloids using a magnet and analyzed with the dynamic light scattering. According to the DLS data, the average hydrodynamic diameter of magnetite alone was around 45 nm, while Fe_3_O_4_/Au nanocomposites with gold content of 1 × 10^−4^–2 × 10^−4^ M were 10–20 nm larger, which corresponded to the size of individual gold NPs (Figure 3a). According to TEM measurements (Figure 3b), the average sizes of gold NPs in the non-magnetic component were of d = 9.1 ± 1.2 nm (1 × 10^−4^ M), 8.7 ± 1.4 (1.5 × 10^−4^ M), 10.6 ± 2.8 (2 × 10^−4^ M).

Further increase in the Au concentration resulted in an increase of the polydispersity index (PDI) for nanocomposites. Specifically, PDI magnitudes were 0.16 for the magnetite sample, 0.18–0.19—for Fe_3_O_4_/Au samples with 1 × 10^−4^…2 × 10^−4^ M, 0.28—for 2.25 × 10^−4^ M, and 0.39 corresponded to 2.5 × 10^−4^ M. Despite this, the average sizes of gold NPs according to TEM measurements were found to remain roughly around 10 nm (d = 8.9 ± 1.5 (2.25 × 10^−4^ M), 9.5 ± 2.4 (2.5 × 10^−4^ M), similarly to the previous series with the same gold content.

**Figure 4:**
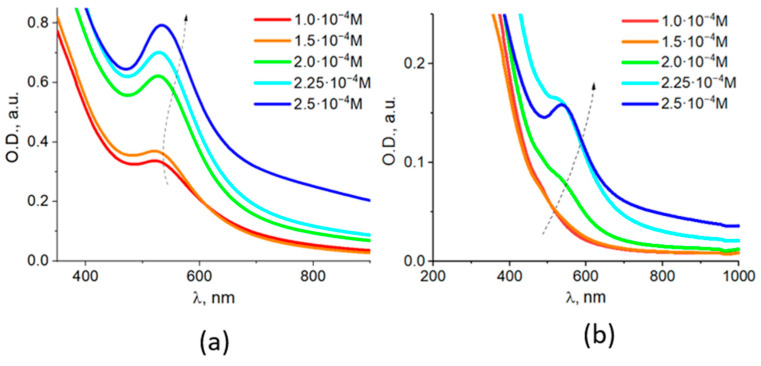
Optical absorption spectra of Fe_3_O_4_/Au nanocomposites prepared by photochemical method. Absorption spectra of Fe_3_O_4_/Au colloids (**a**), and its magnetic fraction separated by a magnet (**b**).

The intensity of the LSPR band of Au in photochemically prepared colloids increased with the increase in metal content in the whole range from 1 × 10^−4^ to 2.5 × 10^−4^ M with the position of the band maxima shifted from 520 to 533 nm. At the highest concentration of Au (2.5 × 10^−4^ M), the plasmonic band became broader with increased absorption in the region of longer wavelengths (Figure 4). 

The LSPR absorption bands of Au in the magnetic Fe_3_O_4_/Au component appeared as a shoulder, forming a band maximum at 535 nm at the highest concentration of Au of C_Au_ = 2.5 × 10^−4^ M. 

**Figure 5:**
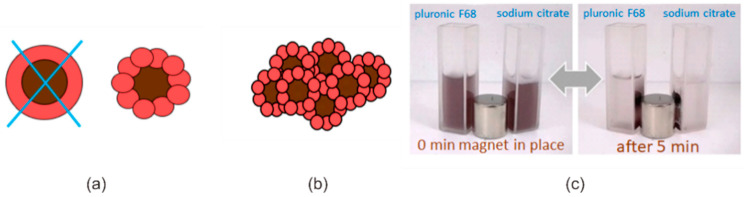
Fe_3_O_4_/Au nanocomposites. Proposed structure of Fe_3_O_4_/Au nanocomposites as a core-satellites (**a**) and their reversible magnetic field-induced assemblies (**b**). (**c**) Illustration of the magnetophoretic effect of Fe_3_O_4_/Au nanocomposites redispersed with pluronic F68 (left cuvette) and sodium citrate (right cuvette) before and after 5 min exposure under magnet.

Based on the data obtained by optical spectroscopy, XPS, TEM and DLS, we propose the structure of magneto-plasmonic nanosystems as nanocomposites with an inhomogeneous gold shell composed of individual NPs attached to the magnetite surface (Figure 5a). According to the classification of magneto-plasmonic nanostructures in [21], these are core-satellite structures. It is important to note that core-satellite gold nanostructures have been recently shown to have higher plasmonic heating efficiency [32], which is beneficial for PTT application.

As the charge of nanoparticles is the key point for the formation of nanocomposite structures, an acid (e.g., nitric acid) can be used to reduce the negative charge of individual gold NPs and stimulate their assembling on the surface of magnetite. In such a way, the gold content and Au/Fe_3_O_4_ ratio in colloids can be controlled, all remaining gold NPs in the non-magnetic fraction can finally be attached to the surface of the magnetic component. 

It should be noted that for both studied series of samples, an increase in gold content (from HAuCl_4_ chloroauric acid used in the synthesis) is accompanied by a simultaneous increase in acid content. As a result, excessive protons compensate the negative charge of gold NPs formed in the alkaline medium, causing their aggregation. At the same time, the zeta potential values for chemically and photochemically prepared Fe_3_O_4_/Au samples (C_Au_ = 2.5 × 10^−4^ M) were found to be −22.2 mV and −31.2 mV, correspondingly. To assess the effect of further acid addition, a drop of nitric acid (C = 0.001 N) was added to both chemically and photochemically prepared colloids. Upon the addition, zeta potential of MPNC was changing –from −22.2 to −6 mV and from −31.2 to −14 mV for the chemically and photochemically obtained Fe_3_O_4_/Au samples, respectively (C_Au_ = 2.5 × 10^−4^ M). Such a decrease in zeta potential allowed for the stronger interaction of magneto-plasmonic and individual plasmonic particles, which can lead to stronger aggregation and formation of larger assemblies. 

**Figure 6:**
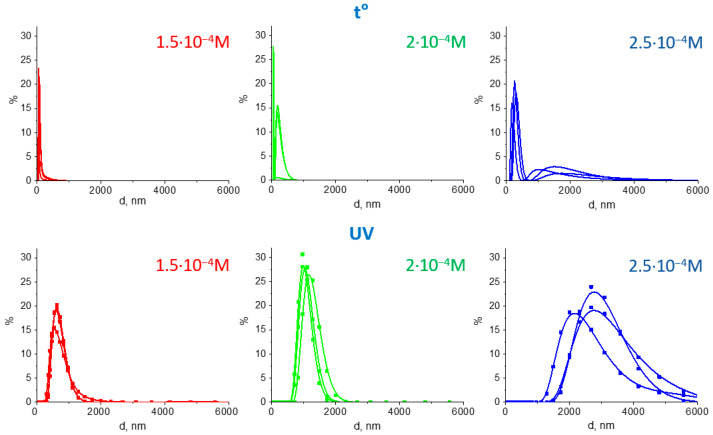
DLS of Fe_3_O_4_/Au nanocomposites. DLS size distribution for the Fe_3_O_4_/Au nanocomposites prepared by the chemical (**top**) and photochemical (**bottom**) methods.

The size of assemblies in chemically prepared samples was found to be up to 500 nm, according to DLS results (Table 2, Figure 6). In the case of an aggregated MPNC (C_Au_ = 2.5 × 10^−4^ M), there was a small fraction of assemblies with sizes of 1–3 μm.

The associates of photochemically synthesized MPNC were several times larger than those in case of chemically synthesized ones (average diameter of 2–4 μm versus 400–1000 nm, correspondingly). This can be advantageous for further activation of the Au-associated photothermal effect [26]. 

**Figure 7:**
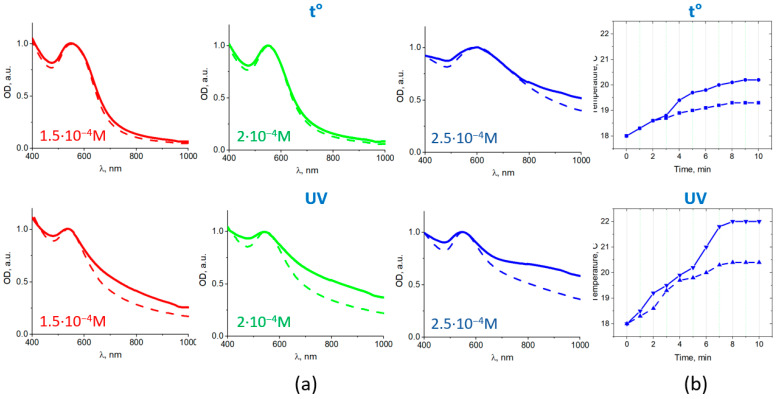
Magnetic field induced changes in absorption spectra of Fe_3_O_4_/Au nanocomposites. Normalized absorbances of Fe_3_O_4_/Au nanocomposites (**a**) obtained by the chemical (**top**) and photochemical (**bottom**) methods before (dashed curves) and after (solid curves) magnet application; photothermal effect (temperature kinetics for the irradiated MPNC suspensions) under NIR (808 nm) laser irradiation of Fe_3_O_4_/Au nanocomposites (C_Au_ = 2.5 × 10^−4^ M) (**b**), prepared by the chemical (**top**) and photochemical (**bottom**) methods in the presence of magnetic field (solid curves) or without it (dashed curves).

The normalized UV–VIS spectra of the colloidal systems after acid addition are shown in Figure 7a (dotted curves). All spectra contained Au plasmonic bands with maxima shifted to the long wavelength region in comparison with those in the initial colloids. The shift was about 17–18 nm for the photochemically prepared samples and 20–35 for the chemically prepared samples (Table 2). All photochemically obtained MPNC had a pronounced NIR absorption, while for chemically obtained systems a pronounced NIR absorption could be noticed only for samples with the highest content of gold.

The magnetic field induced the aggregation of MPNC (as shown in Figure 5c), which resulted in a significant change in absorption. The normalized spectra are shown on Figure 7a (solid curves). The rise of absorption intensity occurred in a broad NIR spectral range without a pronounced peak. This can be explained by a variety of the Au nanocomposites formed, interaction of nanoparticles of different sizes, and formation of assemblies of different number of nanocomposites. For the chemically obtained sample with maximum gold content, the absorption intensity increase in NIR region (at ~800–900 nm) was about 10–20%, while for all photochemically-obtained MPNC it was as high as 40–60%.

Comparing the two series with a similar content of gold, one can conclude that the photochemical method is advantageous for the preparation of Fe_3_O_4_/Au MPNC with the most pronounced reversible optical changes in the NIR region.

Based on the obtained results, the photochemical method and gold content of C_Au_ = 2.5 × 10^−4^ M was chosen as the dynamic MPNC for further application as PTT agent, as there was the most advantageous combination the size of formed assemblies and increase of intensity in the NIR spectral region. Nanosized Fe_3_O_4_/Au composites were collected around the pole of the magnet from a volume of 2 mL with C_Au_ = 2.5 × 10^−3^ M during five minutes for both systems. After washing, such MPNC can be easily redispersed in water using commonly used stabilizers, pluronic F68 or– sodium citrate (Figure 5c). 

### 3.2. Photothermal Effect of Fe_3_O_4_/Au Nanocomposites

An irradiation by the laser diode at 808 nm was found to result in the increase in the temperature of the MPNC suspensions (i.e., photothermal effect). Figure 7b shows temperature kinetics for the MPNC suspensions [C_Au_ = 2.5 × 10^−4^ M, prepared by the chemical (top) and photochemical (bottom) methods] under NIR (808 nm) laser irradiation with power density of 70 mW/cm^2^. It should be noted that the temperature of MPNC suspensions raised higher in the presence of MF (solid curves in Figure 7b) than without it (dotted curves) and there is a correlation between MF induced increase in NIR absorption (Figure 7a) and temperature kinetics in the irradiated MPNC suspension (Figure 7b).

**Figure 8:**
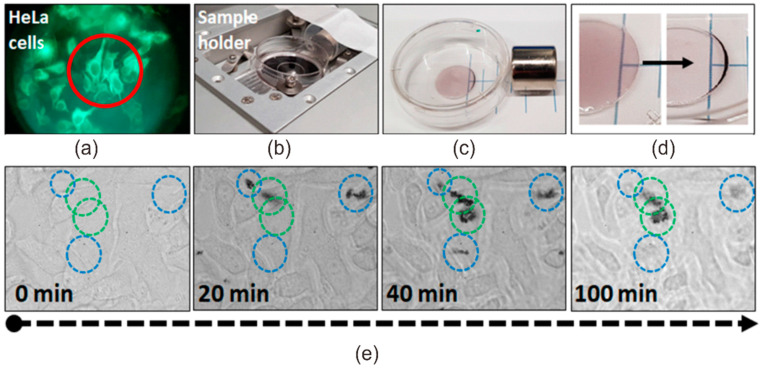
Treatment of HeLa cells with Fe_3_O_4_/Au nanocomposites. Epifluorescence microscopy image (photo acquired through a microscope eyepiece) of HeLa cells incubated with MPNC (**a**); photograph of the microscope sample holder with the cell dish and magnet (**b**); photograph of MPNC suspension on a Petri dish exposed to magnet (**c**); illustration of magnetophoretic movement of MPNC revealed during 15 min (**d**); time series of transmission microscopy images of HeLa cells treated with Fe_3_O_4_/Au nanocomposites (**e**), showing movement of Fe_3_O_4_/Au nanocomposites in the magnetic field (time dependent appearance and disappearance of the aggregated MPNC in regions of interests marked as blue and green circles).

The interaction of magneto-plasmonic Fe_3_O_4_/Au nanocomposites with HeLa cells was studied using confocal laser scanning microscopy in transmission, reflection and fluorescence channels. The Fe_3_O_4_/Au nanocomposites were added to HeLa cells in a dish that was placed in a microscope sample holder with magnet and incubated for 2 h (Figure 8a,b). The cells threated with MPNC were imaged in time using different confocal microscopy modes that provided a complementary information about processes in the cells/MPNC system.

As it is shown in Figure 5c and Figure 8c,d, Fe_3_O_4_/Au nanocomposites move under applied magnetic field. Similarly, MPNC move through the cell medium in the cell dish, being dragged by magnetic field (Figure 8e). Such a behavior of magnetic nanoparticles in cell dish treated with external magnet was previously reported [39]. At the same time, MPNC were found to accumulate on the surface of HeLa cells and remain in the imaged area (Figure 8e, green circles). Moreover, while some MPNC were observed to change their location in time, others did not move, suggesting stronger association with cell surface or cellular uptake. 

To assess the photothermal effect of Fe_3_O_4_/Au nanocomposites in vitro, treated HeLa cells were irradiated with a 593 nm laser. Laser scanning was performed in the same area as for acquisition of the fluorescence or reflection images, which was done using a lower power 485 nm laser. The laser wavelength of 593 nm was closer to the plasmon resonance peak, causing the MPNC to easily absorb the irradiation energy.

**Figure 9:**
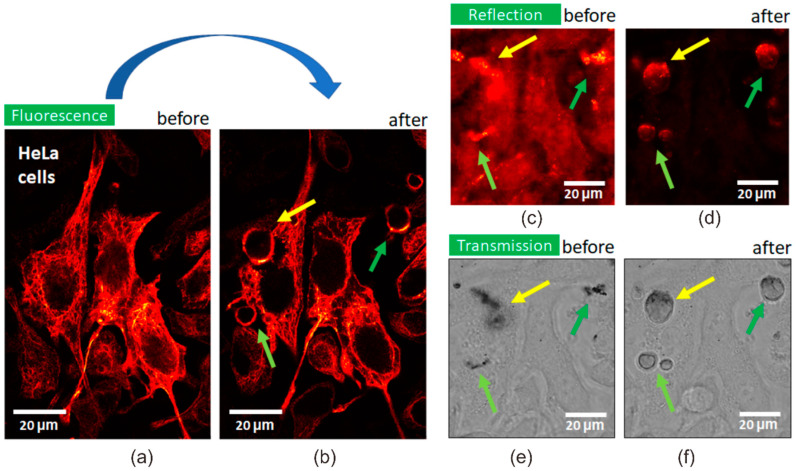
Assessment of photothermal effect of Fe_3_O_4_/Au nanocomposites. Confocal microscopy images ((**a**,**b**) fluorescence, (**c**,**d**) reflection, and (**e**,**f**) transmission) of the formation of microbubbles in HeLa cells treated with Fe_3_O_4_/Au nanocomposites before (left (**a**,**c**,**e**)) and after (right (**b**,**d**,**f**)) irradiation with 593 nm laser.

Obvious changes were observed in cells after irradiation and associated photothermal effect. Instead of the assemblies of nanoparticles on the cell surfaces, which were clearly identified in reflection and transmission modes (Figure 9c,e), the irradiation induced microbubbles on the cell surface (Figure 9b,d,f) through rearrangement of keratin-GFP proteins from fibril structures, typical for keratin filaments, to uniform thin layers of the protein on a rim of microbubbles formed. 

A similar microbubble formation was shown for NIR light irradiated epithelial cells derived from cancerous lung tissue in the presence of gold NPs and 4T1 cell derived solid breast cancer tumors [40,41]. A similar effect was also shown in [26], where phospholipid-based nanomicelles comprising gold nanorods and Fe_3_O_4_ nanoparticles were described. After cellular uptake of the magneto-plasmonic formulation enhanced by the external magnetic field, the laser irradiation at the wavelength of the gold nanorod absorption peak was performed and microbubbles were efficiently generated within cancer cells, causing cell destruction. It is worth, however, noting that no magnetic field induced change in NIR absorption was revealed for this magneto-plasmonic nanoformulation [26]. 

The irradiation/magnetic field induced cavitation regions evidenced the photothermal effect of synthesized magneto-plasmonic nanocomposites causing cytoskeleton and cell membrane damage, which can further cause cell death [26]. The size of bubbles was found to correlate with the size of the irradiated MPNC assemblies: The larger assembly was, the bigger bubble appeared. Microbubbles generated by nanosecond laser pulses were long lived, which allowed us to visualize them in HeLa cells suspension 20 min and more after irradiation. It should be noted that even just cell surface attached but not completely cell-internalized MPNC caused cell damage through a membrane destruction. A rapid magnetophoretic response of the developed MPNC can also allow for their use for targeted drug delivery induced by an applied magnetic field.

## 4. Conclusions

In summary, we developed Fe_3_O_4_/Au magneto-plasmonic nanocomposites with core-satellites structure using tryptophan for metal ion reduction. Owing to the surface plasmon resonance of Au counterparts, the MPNC exhibit well-shaped absorption band in the visible spectral region (540–600 nm) in MPNC. At the same time, because of the presence of Fe_3_O_4_ constituent MPNC can be magnetophoretically-controlled and concentrated using the external magnetic field. A magnetically-induced formation of the MPNC aggregates leads to an increase in optical absorption in NIR spectral rage (800–1000 nm), which can be eliminated by removing the magnetic field. The photochemical method of Fe_3_O_4_/Au MPNC preparation is found to be beneficial for the magnitude of the reversible (induced by presence of magnetic field) optical changes in the NIR region. The in situ magnetically induced aggregation of MPNC, which is accompanied by an increase in NIR absorption, suggests an approach to the magnetic field enhanced NIR-activated photothermal therapy of cancer and other diseases, as it was illustrated by the magnetic field enhanced photothermal effect in MPNC colloids. Furthermore, the magnetic field-induced concentration of MPNC on the surface of cultured cancer cells, followed by irradiation of the cells with scanning laser, led to the pronounced photothermal effect, which caused microbubble formation and membrane damage in the cancer cells. Thus, the synthesized magneto-plasmonic Fe_3_O_4_/Au nanocomposites demonstrate high potential towards magnetophoretically-controlled photothermal therapy. An ability to increase NIR absorption in situ on demand in a targeted diseased tissue for the enhanced photothermal effect can be of importance for the field of phototherapy of cancer and other diseases. A significance of the proposed approach will be further revealed using small animal model of cancer.

## Figures and Tables

**Table 1:** Atomic ratio of the elements in Fe_3_O_4_/Au.

C_Au_, M in Colloid (Initial)	Au:Fe in Colloid (Initial)	Au:Fe 2p in Magnetic Component (XPS)
1.0 × 10^−4^	0.667	0.053 (5:95)
1.5 × 10^−4^	1.000	0.111 (10:90)
2.0 × 10^−4^	1.333	0.333 (25:75)
2.5 × 10^−4^	1.667	2.333 (70:30)
3.0 × 10^−4^	2.000	2.333 (70:30)

**Table 2:** Optical and morphological characteristics of Fe_3_O_4_/Au nanocomposites.

Sample	λ_max 0_, nm	λ_max 1_, nm	Δ*λ_H+_*, nm	λ_max 2_, nm	r _800_	r _900_	Fe_3_O_4_/Au Size, nm (DLS)	Gold NPs Size, nm (TEM)
t-1.5	527	549	*22*	549	1.30	1.35	<200	11.8 ± 2.5
t-2.0	532	552	*20*	549	1.22	1.28	100–400	8.9 ± 1,4
t-2.5	567	602	*35*	600	1.07	1.20	200–500 (1000–3000)	10.6 ± 2.7
UV-1.5	523	540	*17*	536	1.45	1.50	400–1000	8.7 ± 1.4
UV-2.0	527	545	*18*	543	1.55	1.66	800–1500	10.6 ± 2.8
UV-2.5	533	551	*18*	548	1.36	1.52	2000–4000	9.5 ± 2.4

## Data Availability

The data presented in this study are available upon request from the corresponding author.
